# Improving risk stratification of PI-RADS 3 + 1 lesions of the peripheral zone: expert lexicon of terms, multi-reader performance and contribution of artificial intelligence

**DOI:** 10.1186/s40644-025-00916-7

**Published:** 2025-08-19

**Authors:** Philip A. Glemser, Nils Netzer, Christian H. Ziener, Markus Wilhelm, Thomas Hielscher, Kevin Sun Zhang, Magdalena Görtz, Viktoria Schütz, Albrecht Stenzinger, Markus Hohenfellner, Heinz-Peter Schlemmer, David Bonekamp

**Affiliations:** 1 Division of Radiology, German Cancer Research Center (DKFZ), Im Neuenheimer Feld 280, 69120 Heidelberg, Germany; 2 Department of Radiation Oncology, Heidelberg University Hospital, Heidelberg, Germany; 3 Heidelberg University Medical School, Heidelberg, Germany; 4 Breast Diagnostics Munich, Sonnenstraße 29, Munich, Germany; 5 Division of Biostatistics, German Cancer Research Center (DKFZ), Heidelberg, Germany; 6 Department of Urology, Heidelberg University Hospital, Heidelberg, Germany; 7 Junior Clinical Cooperation Unit ‘Multiparametric Methods for Early Detection of Prostate Cancer’, German Cancer Research Center (DKFZ), Heidelberg, Germany; 8 Institute of Pathology, University of Heidelberg Medical Center, Heidelberg, Germany; 9 National Center for Tumor Diseases (NCT) Heidelberg, Heidelberg, Germany; 10 German Cancer Consortium (DKTK), Heidelberg, Germany

**Keywords:** Prostate cancer, Risk stratification, Lexicon terms, Deep learning, Extended fused biopsy

## Abstract

**Background:**

According to PI-RADS v2.1, peripheral PI-RADS 3 lesions are upgraded to PI-RADS 4 if dynamic contrast-enhanced MRI is positive (3+1 lesions), however those lesions are radiologically challenging. We aimed to define criteria by expert consensus and test applicability by other radiologists for sPC prediction of PI-RADS 3+1 lesions and determine their value in integrated regression models.

**Methods:**

From consecutive 3 Tesla MR examinations performed between 08/2016 to 12/2018 we identified 85 MRI examinations from 83 patients with a total of 94 PI-RADS 3+1 lesions in the official clinical report. Lesions were retrospectively assessed by expert consensus with construction of a newly devised feature catalogue which was utilized subsequently by two additional radiologists specialized in prostate MRI for independent lesion assessment. With reference to extended fused targeted and systematic TRUS/MRI-biopsy histopathological correlation, relevant catalogue features were identified by univariate analysis and put into context to typically available clinical features and automated AI image assessment utilizing lasso-penalized logistic regression models, also focusing on the contribution of DCE imaging (feature-based, bi- and multiparametric AI-enhanced and solely bi- and multiparametric AI-driven).

**Results:**

The feature catalog enabled image-based lesional risk stratification for all readers. Expert consensus provided 3 significant features in univariate analysis (adj. p-value <0.05; most relevant feature T2w configuration: “irregular/microlobulated/spiculated”, OR 9.0 (95%CI 2.3-44.3); adj. p-value: 0.016). These remained after lasso penalized regression based feature reduction, while the only selected clinical feature was prostate volume (OR<1), enabling nomogram construction. While DCE-derived consensus features did not enhance model performance (bootstrapped AUC), there was a trend for increased performance by including multiparametric AI, but not biparametric AI into models, both for combined and AI-only models.

**Conclusions:**

PI-RADS 3+1 lesions can be risk-stratified using lexicon terms and a key feature nomogram. AI potentially benefits more from DCE imaging than experienced prostate radiologists.

**Clinical trial number:**

Not applicable.

**Supplementary Information:**

The online version contains supplementary material available at 10.1186/s40644-025-00916-7.

## Background

The prostate imaging reporting and data system (PI-RADS) has led to standardization of multiparametric prostate MRI (mpMRI) interpretation and reporting and plays a crucial role for detection and staging of prostate cancer (PC). In the current PI-RADS version 2.1, a PI-RADS lesion in the peripheral zone (PZ) receives a total score of 4 even if the DWI/ADC-subscore is 3, if the dynamic contrast-enhanced MRI (DCE-MRI) is focally positive for early contrast enhancement (PI-RADS 3 + 1 lesion) [1]. However, it has been shown that PI-RADS 3 + 1 lesions carry a lower risk for harboring significant prostate cancer (sPC) than PI-RADS 4 lesions with an DWI/ADC-subscore of 4 [2–4]. Presence of focal enhancement problematically produces a decision between PI-RADS 2 and 4 in situations where there is a marginal range between non-focal and focal findings on the T2w and ADC images. In these cases, differentiation between PI-RADS 2 and PI-RADS 4 (3 + 1) results is challenging in daily practice, leading to unnecessary biopsies or undetected sPC.

The benefit of integrating DCE sequences as part of a multiparametric MRI for prostate imaging overall, as well as in terms of risk stratification of indeterminate ADC-PI-RADS 3 lesions, is subject to growing debate and has not yet been answered [4–13].

In a first attempt to risk-stratifying PZ lesions, Chatterjee et al. showed that a higher percentage of T2w wedge-shaped PZ lesions were malignant than previously assumed and suggested preferring quantitative ADC values for distinguishing malignant vs. benign conditions [14]. In another study with focus on PI-RADS 4 lesions in the PZ, lesion shape according to the PI-RADS v2 lexicon or different patterns of PZ sparing, i.e. normal PZ visible between lesion and prostate capsule (peripheral sparing) or between lesion and transition zone (TZ) (central sparing), were not in general predictive for sPC [2].

Clear sPC feature predictors in the gray area between PI-RADS 2 and 4 are lacking and the definition of a comprehensive feature set for stratification of PI-RADS 3 + 1 lesions is pending. For general PI-RADS lexicon terms the association with sPC, PC or benign prostate changes has been addressed previously [15] and recent results from the use of deep learning in prostate imaging have been promising [16–19]. Nonetheless, additional lexicon terms, clinical features, the discriminatory power of CNN approaches and the standard lexicon terms specifically applied in PI-RADS 3 + 1 lesions remain to be clarified.

While it is important to validate any diagnostic imaging approach in a multi-center, multi-vendor setup or even better in different clinical settings before recommending it for integration into clinical practice, this approach may carry difficulties. Indeed, the value of multi-center, multi-vendor studies lies in the consideration of added data heterogeneity, but this always introduces the challenge of maintaining overall compatible high imaging and reporting quality and complicates statistical analyses as the added variability usually requires larger cohort sizes. In this context, there is justifiable reason to evaluate new ideas and concepts first in a single-center, single-vendor setting. In particular, regarding the challenging issue of PI-RADS 3 + 1 at the threshold of inflammatory and incipient suspicious changes, we assume that a homogeneous single-center, single-vendor study performed in an experienced academic center with long-term prostate imaging expertise and use of high-quality MRI protocols may potentially address the complex issue of lesion interpretation.

We hypothesized that MR-based criteria can be defined that allow stratification of PI-RADS 3 + 1 lesions into subgroups with different sPC risk. The aim of this study was to retrospectively establish a comprehensive feature set by expert consensus review of mpMRI with reference to extended MRI/TRUS-biopsy ground truth and to evaluate the discriminatory power of the feature set in 3 + 1 lesions through application of the defined criteria by two additional experienced prostate radiologists. We also aimed to put the potential added benefit of the derived features into context with typically available clinical features and with automated AI-based image analysis differentiation both, a biparametric (DWI, T2w) and a multiparametric (DWI, T2w, DCE) information context. Based on these assessments, we set out to determine the optimal strategy for risk assessment utilizing a combination of radiological features, clinical features and AI for improved prediction accuracy and simplification of the decision between biopsy and follow-up.

## Methods

### Patient characteristics

This retrospective study was approved by the institutional ethics committee (institutional ethics approval number S-164/2019) and conducted in accordance to the declaration of Helsinki in its current form. Inclusion criteria of patients were as follows: (1) included in prior patient cohort of our group previously published by Schrader et al. [20] (2) examination in the time-frame from 08/2016 to 12/2018 and (3) at least one PI-RADS 3 + 1 lesion call in the initial clinical read. Significant PC was defined according to International Society of Urological Pathology (ISUP) grade of ≥ 2. Patient selection flow chart is given in Supplemental Fig. 1. The patient cohort has been reported in several previous publications dealing with artificial intelligence and prostate cancer prediction [16, 17, 19–23], however, data have not been used in a systematic sub-cohort analysis of PI-RADS 3 + 1 lesions so far.

### MRI protocol

Multiparametric MRI including T2w, diffusion-weighted (DWI) and dynamic contrast-enhanced MRI series (DCE) were performed on a 3 Tesla MRI scanner system (Prisma, Siemens Healthcare, Erlangen, Germany) under usage of the standard multichannel body coil and integrated spine-phase-array coil, based on PI-RADS recommendations [1, 24] and guidelines of the European Society of Urogenital Radiology [25, 26]. Detailed MRI protocol information can be found in Supplemental Material 1.

### Histological correlation with MRI/TRUS-fusion biopsies

All patients underwent sextant-specific systematic extended transperineal MRI/TRUS-fusion biopsies (mean 24.7 cores, IQR 22–28) according to the Ginsburg biopsy scheme as well as fusion-targeted biopsies of suspicious MRI lesions in the initial clinical read with a PI-RADS score ≥ 3 (mean 4.7 cores per lesion, IQR 4.7–6). For details see Supplemental Material S2. This extended biopsy approach at our institutions has showed adequate concordance with radical prostatectomy specimen in previous validations [27].

### PIRADS 3 + 1 lesions: imaging feature set (lexicon of terms) and reassessment of clinically reported lesions in consensus and single reads

**Fig. 1:**
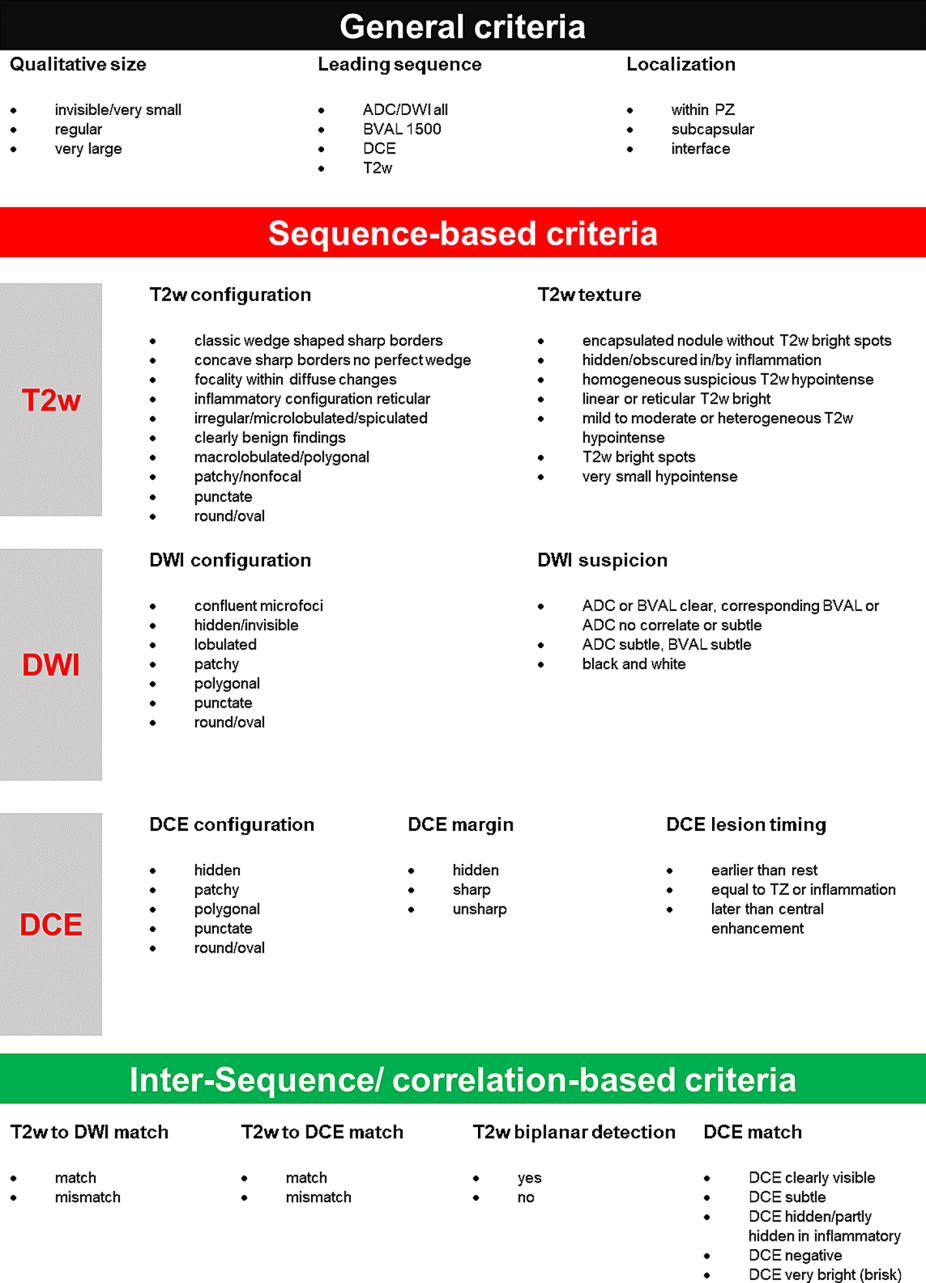
Feature set (lexicon of terms) for risk-stratifying PI-RADS 3 + 1 lesions including general, sequence-based (T2w, DWI and DCE) and inter-sequence/correlation-based feature criteria. For each lesion the best fitting phrase in each category was assigned in the reading sections

Two prostate MRI experts with 14 respectively 8 years of experience in prostate imaging defined a feature set for PIRADS 3 + 1 lesions including general, sequence-based (T2w, DWI and DCE) inter-sequence/correlation-based criteria as mentioned in Fig. 1. For each feature (i.e. qualitative size) several options (lexicon of terms) were phrased and only the best fitting phrase should be assigned. Features are grouped into new and PI-RADS related features according to Supplemental Table 1. All clinically called PI-RADS 3 + 1 lesions were reassessed by consensus read by applying the abovementioned criteria and determining a lesion-based PI-RADS score (2, 3, 3 + 1, 4, 5) to the respective lesion(s) of each patient. In a second step, two board-certified radiologists with > 5 years of experience in prostate imaging were instructed about the feature set and evaluated those lesions independently. All readers were blinded to patients’ clinical and laboratory data.

### Biparametric and multiparametric AI analysis

For each examination, multi-parametric axial images were collected: T2-weighted (T2w), DWI with low and high b-values, ADC maps, and 4D-DCE-MRI images. Prostate masks were generated for T2w, DWI, and DCE-MRI using nnUNet-based custom models for each modality. The earliest contrast appearance in DCE, deemed critical by PI-RADS guidelines, was identified using mean signal intensity within the prostate mask. Four time steps surrounding this “wash-in point” were selected to focus on baseline and early enhancement characteristics of DCE. These images were then prepared as input for neural networks. The collected images and their corresponding prostate masks were resampled to 0.58 × 0.58 × 3 mm dimensions. All images were normalized by subtracting their mean and dividing by the standard deviation, in the case of ADC maps using global dataset statistics. Since only T2w annotations were utilized, precise cross-modality registration was used, with image co-registration as previously described [19]. The Dice coefficient was used to assess the overlap between fixed T2w and moving DWI/DCE prostate masks. If the overlap was suboptimal (< 0.70), automatic rigid alignment using the masks’ centers of mass was initiated. Training was conducted with a batch size of 6 over 1000 epochs, each containing 250 batches, in a five-fold cross-validation setup. Lesion segmentation networks were created for bpMRI and mpMRI using nnUNet, accommodating either bi-parametric four-parameter (T2w, low- and high b value, ADC map) or 8-parameter multi-parametric (bi-parametric plus four DCE timepoints) inputs. Training and calibration were performed on a dataset of *N* = 1456, excluding the examinations used for 3 + 1 lesion assessment in this study. After training, inference was performed on the current dataset, and the CNN prediction probability of sPC determined as the maximum softmax value within the manually segmented 3 + 1 lesion mask (denoted as *lesion-max*).

### Statistical analysis

Descriptive statistics of the cohort were calculated and tabulated. In a first step, univariable analysis of feature categories was performed, and evaluated using Fisher’s exact test (p-value two-sided). Subsequently, univariate analysis of individual features was performed using Fisher’s exact test (p-value, two-sided). For this analysis, individual features were pre-selected, keeping only those with a prevalence between 10% and 90% and any pairwise correlation below 0.8. Multiple testing adjustment was applied controlling the false discovery rate. The global test approach according to Goeman [28] using a logistic model was applied for identifying potential groups of individual features associated with sPC. To further select relevant features, we performed lasso-penalized logistic regression choosing lambda 1SE (standard error) as penalty based on 20-fold cross-validation.

The ROC AUC performance of lasso penalized logistic regression, with respect to sPC association, was evaluated using 4 different models and we also evaluated the biparametric and multiparametric softmax output individually. Each regression also included the following clinical variables/features: “PSA”, “patient’s age”, “histological results of any previous biopsies” (none versus no malignancy versus Gleason 6) and “log-transformed prostate volume” as potential predictors. This resulted in the following models: *model 1* (all pre-selected consensus read features + clinical features), *model 2* (all pre-selected consensus read features excluding DCE features + clinical features), *model 3* (model 2 + biparametric convolutional neural network (bpCNN) lesion-max), *model 4* (model 1 + multiparametric CNN (mpCNN) lesion-max) as well as *solely biparametric CNN* (bpCNN lesion-max) and *multiparametric CNN* (mpCNN lesion-max) as mentioned above. Model performance was assessed based on bootstrapped ROC-AUC. Inter-rater agreement was assessed using Cohen’s weighted kappa. Analyses were performed with software R using add-on packages rms, glmnet and riskRegression.

## Results

### Study population

Of 673 MR examinations within the inclusion period between August 2016 and December 2018 followed by biopsy at our institutions, 85 examinations in 83 patients (median patient age:64, 45–85 years) were included in the study. Of those patients, 56 (67.5%) were biopsy-naïve, 27 (32.5%) received prior biopsies with evidence of Gleason 6 in 10 patients (12%) and 18 (22%) were enrolled in active surveillance. 83 patients in the study sample had 94 PI-RADS 3 + 1 lesions according to the initial clinical report. 27 of 94 lesions (29%) were positive for sPC at biopsy. Demographic data and patient characteristics are given in Table 1.

**Table 1:** Demographic and clinical characteristics of 83 included men

Variable	Cohort *n* = 83 patients
Age (years) median (IQR)	64 (58–69)
PSA (ng/ml) median (IQR)	7.4 (5.0–10.0)
PSA density median (IQR)	0.1 (0.1–0.2)
Timing (days) between mpMRI and biopsy median (IQR)	1 (1-6.25)
ISUP grading of PIRADS 3 + 1 lesions (*n* = 94) PER LESION	
No cancer	48
ISUP 1	19
ISUP 2	19
ISUP 3	4
ISUP 4	2
ISUP 5	2
No sPC	67
sPC	27

Abbreviations PSA = prostate-specific-antigen, ISUP = International Society of Urological Pathology, sPC = clinically significant prostate cancer

### Correlation of lesion-based PI-RADS scores by different readers with extended biopsy ground-truth

As stated in Table 2 the expert consensus read resulted in a lesion-based sensitivity and specificity of 96.3% and 26.9% for PI-RADS threshold ≥ 3, 92.6% and 31.3% for PI-RADS threshold ≥ 3 + 1 and 48.1% and 89.6% for PI-RADS threshold ≥ 4. This results in one false-negative lesion for PI-RADS threshold ≥ 3 and two false-negative lesions for PI-RADS threshold ≥ 3 + 1 in the consensus read. The assessment of additional Reader 1 (R1) showed lower sensitivity and higher specificity with 40.7% and 77.6% for PI-RADS threshold ≥ 3, 40.7% and 80.6% for PI-RADS threshold ≥ 3 + 1 and 29.6% and 89.6% for PI-RADS threshold ≥ 4. This yielded 16 falsely negative evaluated lesions each for PI-RADS threshold ≥ 3 and ≥ 3 + 1. The reading assessment of additional single reader 2 showed slightly lower sensitivity as well as partially higher specificity compared to consensus read with 85.2% and 34.3% for PI-RADS threshold ≥ 3, 81.5% and 41.8% for PI-RADS threshold ≥ 3 + 1, and 37 and 70.1% for PI-RADS threshold ≥ 4. Four and accordingly five false negative lesions resulted here for PI-RADS threshold ≥ 3 and PI-RADS threshold ≥ 3 + 1.

**Table 2:** Lesion-based diagnostic reader performance (consensus read vs. single-reads) against extended targeted and systematic MRI/TRUS-biopsy histopathology ground truth. Statistical evaluation of reading section under regard of PI-RADS thresholds ≥ 3, ≥3 + 1 and ≥ 4

	PI-RADS threshold	Sensitivity	Specificity	tn	tp	fn	fp
Consensus read	≥ 3	96.3	26.9	18	26	1	49
	≥ 3 + 1	92.6	31.3	21	25	2	46
	≥ 4	48.1	89.6	60	13	14	7
Single reader 1	≥ 3	40.7	77.6	52	11	16	15
	≥ 3 + 1	40.7	80.6	54	11	16	13
	≥ 4	29.6	89.6	60	8	19	7
Single Reader 2	≥ 3	85.2	34.3	23	23	4	44
	≥ 3 + 1	81.5	41.8	28	22	5	39
	≥ 4	37.0	70.1	47	10	17	20

Abbreviations: tn = true negative, tp = true positive, fn = false negative, fp = false positive

### Inter-rater agreement

Inter-rater agreement according to Cohen’s kappa between consensus and R1 was 0.2 (slight to fair), between consensus and R2 0.26 (fair) and between R1 and R2 0.22 (fair). As shown in Supplemental Tables 2–4, this corresponds to identical PI-RADS decisions between consensus and R1 in 43% of lesions, between consensus and R2 in 62% of lesions and between R1 and R2 in 47% of lesions.

### Univariable analysis of feature categories

In the univariate analysis of feature categories based on the consensus read, the categories “DWI suspicion”, “T2w configuration”, “T2w texture”, “DWI configuration” and “qualitative size” were significantly associated with sPC (adj. p-value, all *p* = 0.041); while the other categories, including “DCE lesion timing (p = 0.058)”, “leading sequence (p = 0.101)”, “DCE match (p = 0.161)”, “DCE configuration (p = 0.214)”, “T2w to DWI match (p = 0.302)”,“DCE margin (p = 0.432)”, “T2w biplanar detection (p = 0.481)”, “localization (p = 0.894)” and “T2w to DCE match (p = 1.0)” were not.

### Univariable analysis of individual features

Univariable analysis of individual features from the consensus read before multiplicity adjustment (see Supplemental Table 5) identified eight features demonstrating significant effects with regard to sPC prediction. The significant lesion parameters (uncorrected p-value ≤ 0.05) were “T2w configuration irregular/microlobulated/spiculated (T2cims)” (*P* < 0.001), “T2w texture homogeneous suspicious T2w hypointense (T2thsT2h)” (*p* = 0.003), “DWI suspicion black and white (DWIbaw)” (*p* = 0.004), “DWI suspicion ADC subtle/BVAL subtle (DWIsADCsBVALs)” (*p* = 0.009), “leading sequence T2w (lsT2)” (*p* = 0.012), “DCE lesion timing earlier than rest (DCEltetr)” (*p* = 0.018), “T2w texture linear or reticular T2w bright (T2tlorT2b)” (*p* = 0.02) and “T2w configuration concave sharp borders no perfect wedge (T2ccsbnpw)” (*p* = 0.03). After subsequent multiplicity adjustment, three features remained significant: “T2cims” (adj. *p* = 0.016) and subordinate “T2thsT2h” and “DWIbaw” (adj. *p* = 0.046 each).

### Global test approach

**Fig. 2:**
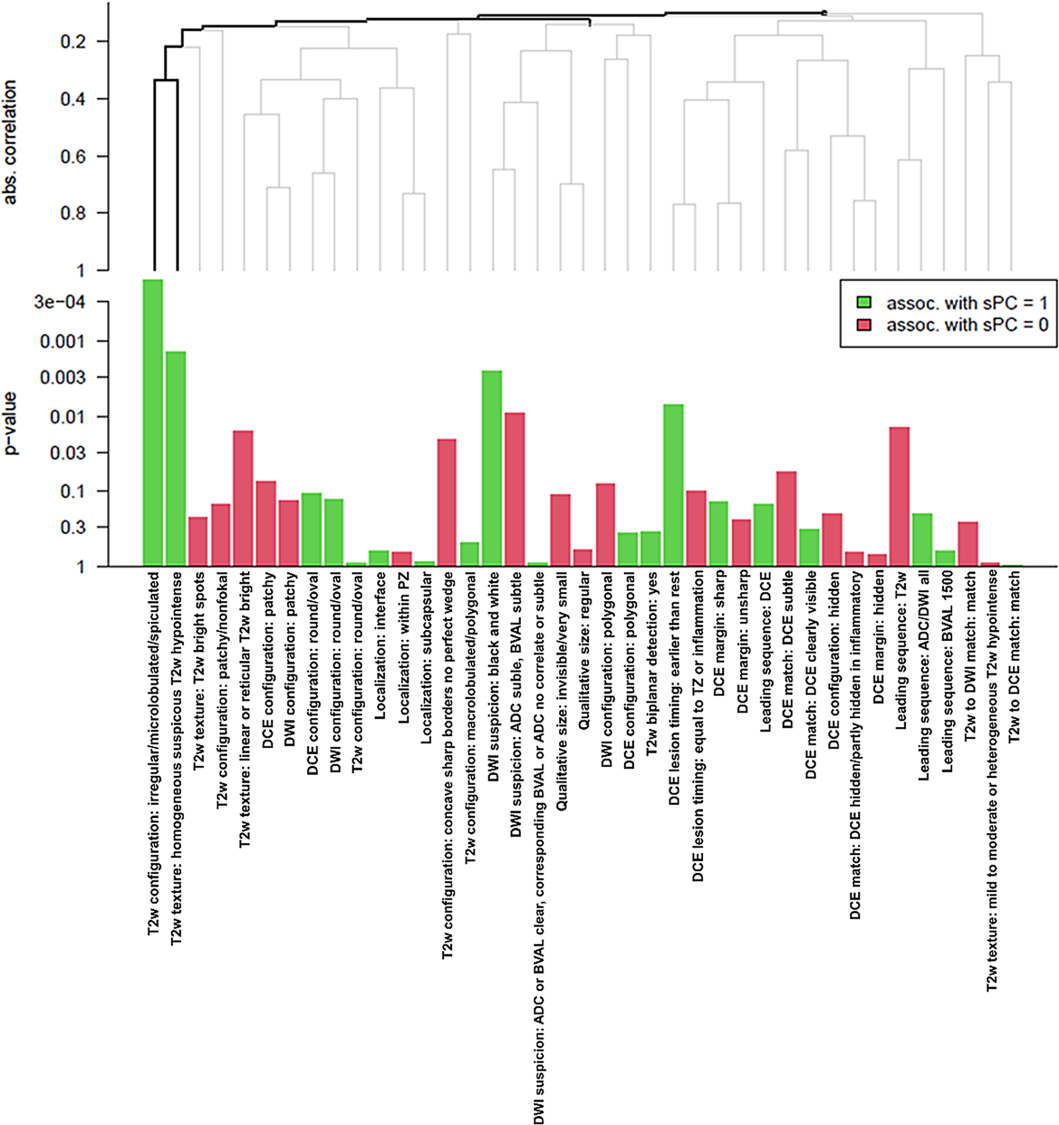
Bar chart for selected radiological feature contribution (positive association with sPC in green, negative in red) based on the Goeman test. 4 features each were significantly associated with sPC (p-value < 0.05; “T2w configuration irregular/ microlobulated/ spiculated”, “T2w texture homogeneous suspicious T2w hypointense”, “DWI suspicion black and white” and “DCE lesion timing earlier than rest”) and with non sPC in descending order (“DWI suspicion ADC subtle/BVAL subtle”, “leading sequence T2w”, “T2w texture linear or reticular T2w bright” and “T2w configuration concave sharp borders no perfect wedge”). A significant cluster following multiple-testing adjustment of the individual features “T2w configuration irregular/microlobulated/spiculated” and “T2w texture homogeneous suspicious T2w hypointense” was found.

Using Goeman’s global test [28] (see Fig. 2) the parameters “T2cims”, “T2thsT2h”, “DWIbaw” and “DCEltetr” were associated with sPC by decreasing order. Otherwise, the parameters “DWIsADCsBVALs”, “lsT2”, “T2tlorT2b” and “T2ccsbnpw” revealed association with non sPC by decreasing order. As such, there was mostly agreement between the individual feature contribution and the univariable analyses with uncorrected p-values above. When applying multiple testing correction, a significant cluster consisting of “T2cims” and “T2thsT2h” was identified.

### Lasso penalized regression models

Lasso penalized logistic regression models enabled the consideration of clinical features and the reduction of the extensive consensus-based feature models to just a few features (see Table 3) and underlined the relevance of the feature “T2cims” that remained the first-ranked expert consensus-based feature in all models according to the Odds Ratio. The features “T2thsT2h“ and “DWIbaw” were represented subordinately in all feature-based (model 1 and 2) or feature-based/AI assisted models (model 3 and 4). In model 3, the feature “bpCNN lesion-max” was not selected and the features “T2thsT2h” and “DWIbaw” revealed comparatively small model coefficients. In model 4, with a bootstrapped ROC AUC performance of 0.71, multiparametric CNN (mpCNN lesion-max) was selected and another feature “lsT2” emerged fifth-ranked and negatively correlated with sPC (OR < 1). Of all clinical features, only the feature “log-transformed prostate volume” was selected subordinately and negatively correlated with sPC (OR < 1) in model 1–4.

**Fig. 3:**
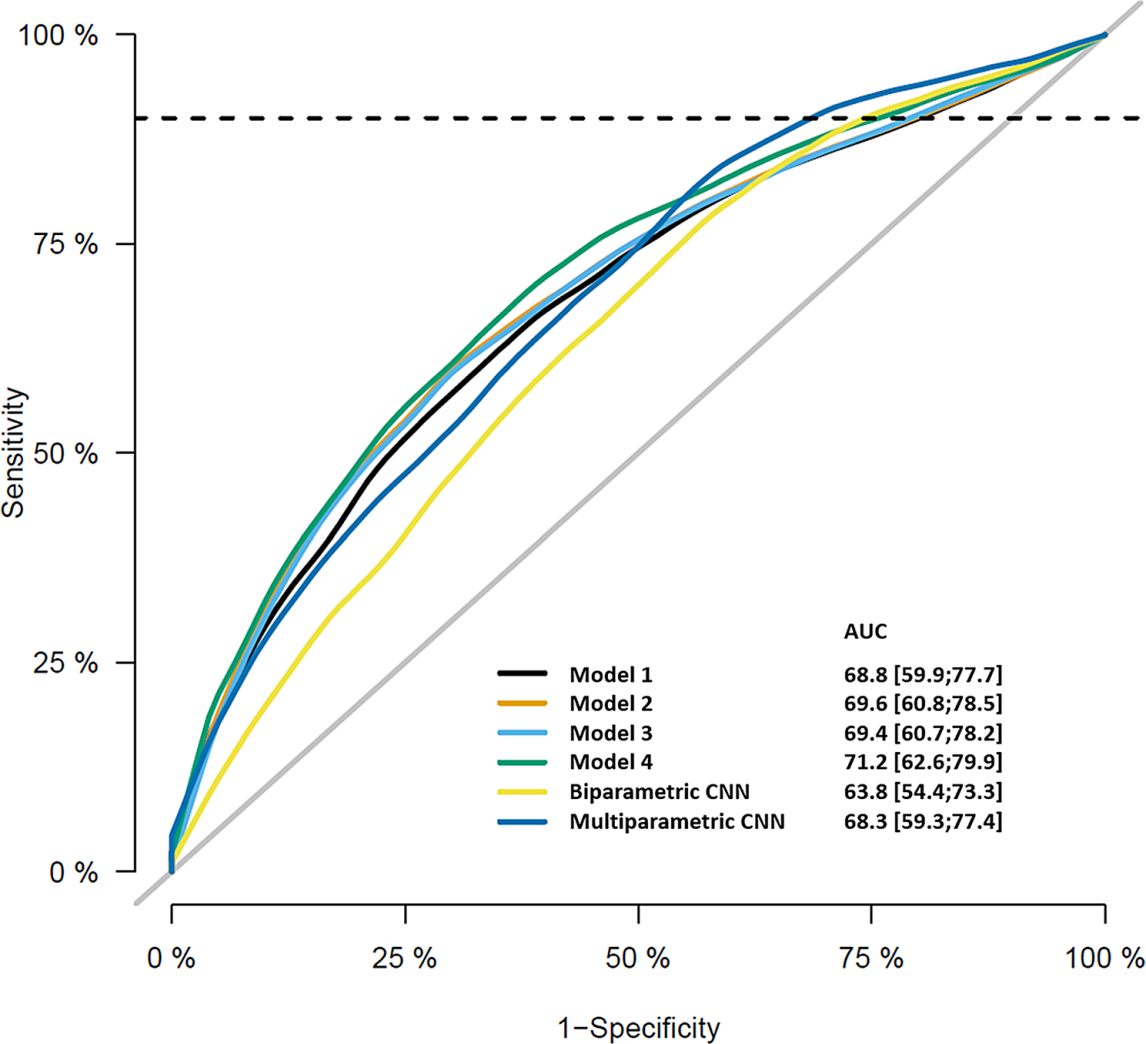
Receiver operating characteristic (ROC) curves averaged across bootstrap runs are shown for lasso-penalized logistic regression models 1–4 as well as for solely AI-driven biparametric and multiparametric CNNs (AUC values with corresponding 95% confidence intervals in brackets). Based solely on the consensus read and clinical features, biparametric model 2 even showed a slightly better performance than the multiparametric model 1, indicating no inferiority of the model that excluded all DCE features. AI-assisted biparametric (model 3) and multiparametric (model 4) approaches showed a slight advantage for model 4 with 0.71 vs. 0.69 in terms of AUC while a more pronounced benefit was observed for the solely AI-driven multiparametric CNN compared to the biparametric CNN with 0.68 vs. 0.64. Abbreviations: AUC = area under the curve

**Fig. 4:**
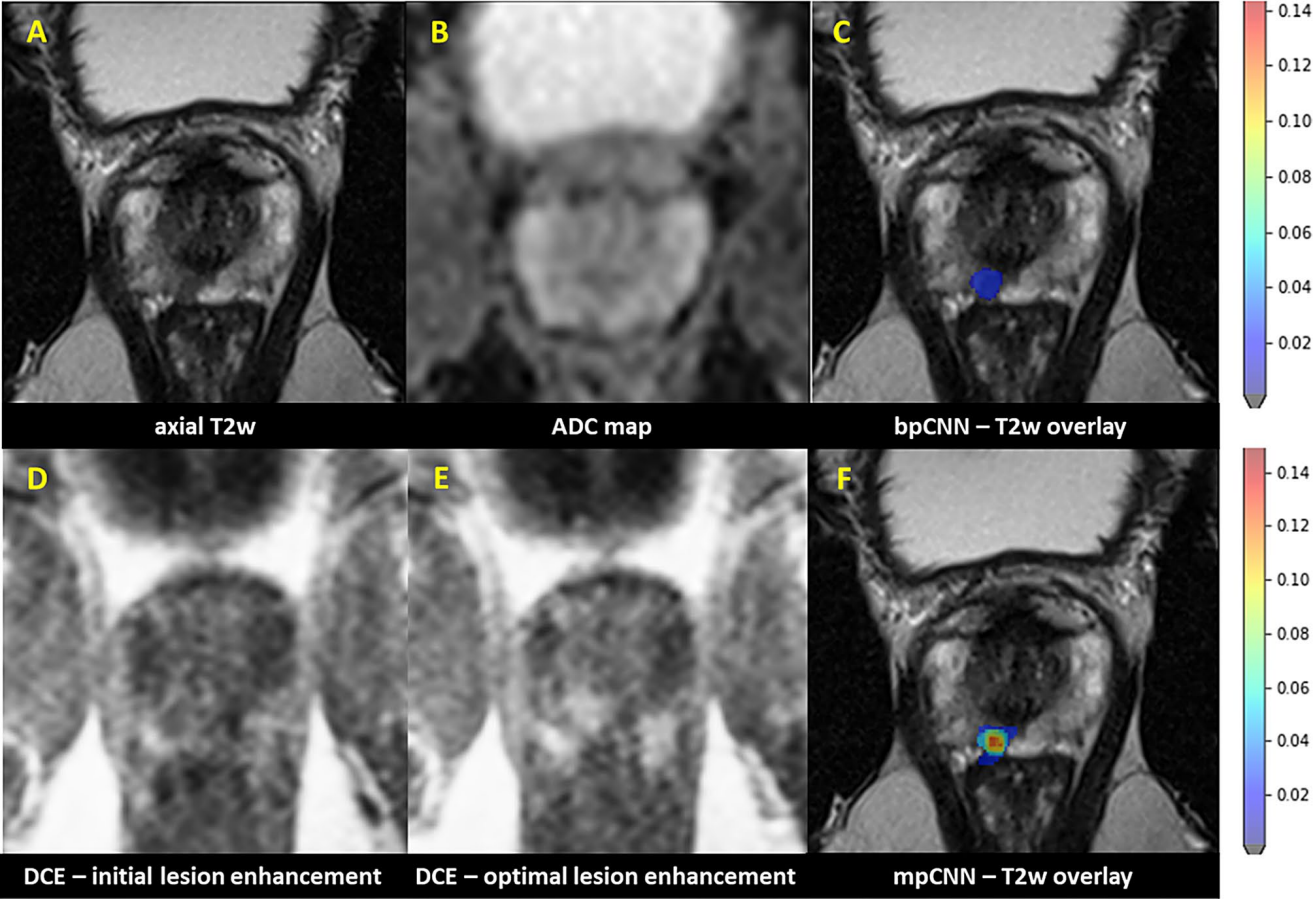
Histology-confirmed an ISUP 2 carcinoma. 45 year-old patient with PSA-elevation (5.7 ng/ml, PSA density: 0.31 ng/ml^2^); clinical lesion assessment: PI-RADS 3 + 1 = 4 in the posteromedial peripheral zone (PZpm) in the right apex. This focal lesion revealed T2w hypointense (**A**), with an irregular and partially microlobulated/spiculated T2w configuration and subtle diffusion restriction with signal decrease in the corresponding ADC-map (**B**). In dynamic acquisition (DCE) a distinct early focal enhancement in comparison of initial (**D**) and optimal enhancement phase (**E**) was shown. In expert consensus read it was also classified as PI-RADS 3 + 1 = 4. AI probability score aligns well with the MRI findings and depicts a high probability score for PCa (DL-PI-RADS 4) in bi- and multiparametric CNN (**C** and **F**; axial T2w with respective CNN overlay) with a higher sPC probability score in the DL-PI-RADS 4 continuum for mpCNN.

**Fig. 5:**
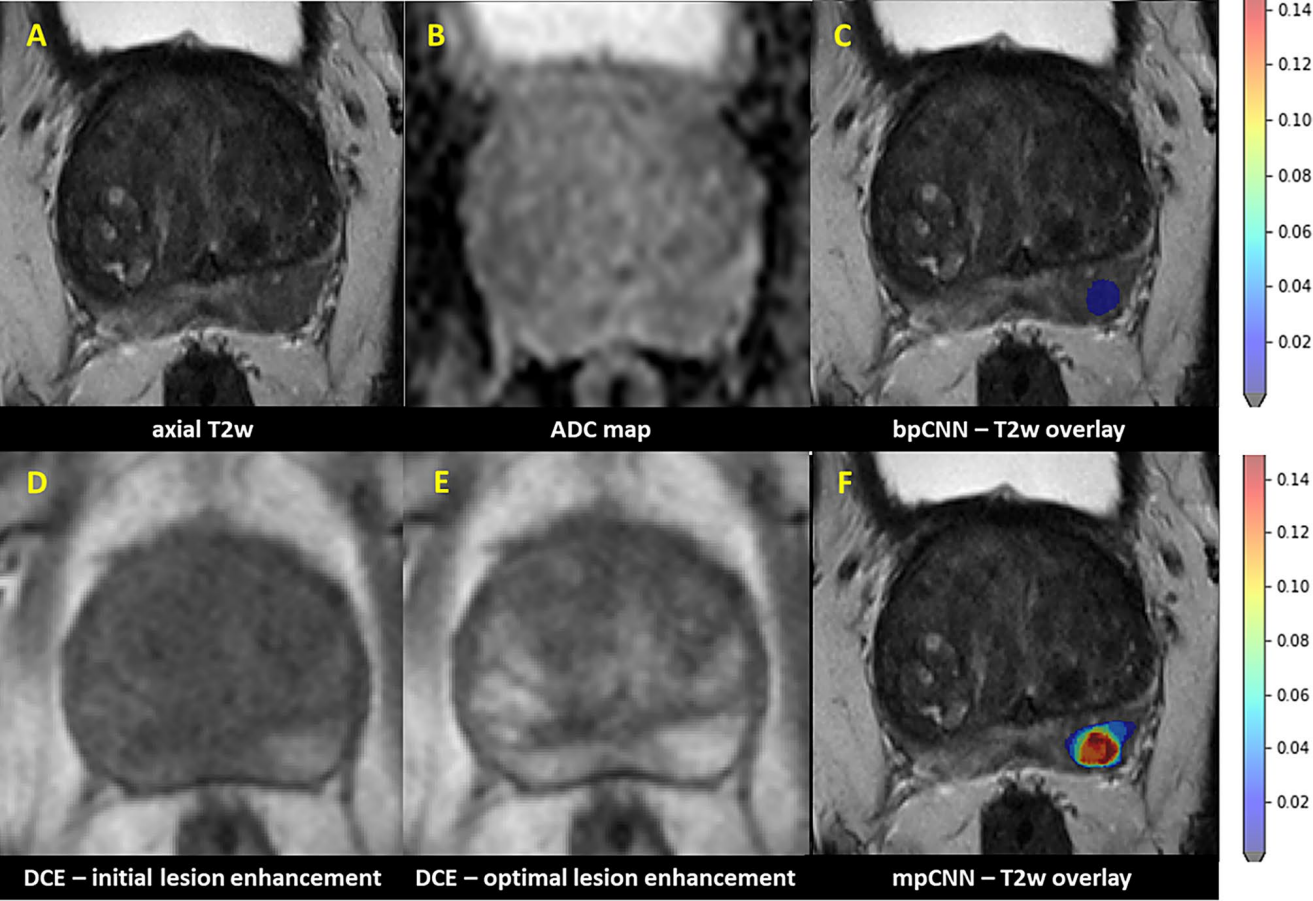
69-year old patient with PSA-elevation (18 ng/ml, PSA density: 0.18 ng/ml^2^); clinical lesion assessment: PI-RADS 3 + 1 = 4 in the left posteromedial/-lateral peripheral zone in the prostate mid. This focal lesion revealed patchy/macrolobulated with a suspicious homogeneous T2w hypointense T2w texture (**A**) and a subtle diffusion restriction with slight signal decrease in the corresponding ADC-map (**B**). In dynamic acquisition (DCE) a clear early focal enhancement in the initial enhancement phase (**D**) was shown, in optimal enhancement phase (**E**) the surrounding PZ shows nonfocal enhancement with the lesion itself appearing more focally accentuated. In expert consensus read it was classified as PI-RADS 3 + 1 = 4. AI probability score fits well with the MRI findings and shows a high probability score for PCa (DL-PI-RADS 4) in bi- and multiparametric CNN (**C** and **F**; axial T2w with respective CNN overlay) with a higher sPC probability score in the DL-PI-RADS 4 continuum for mpCNN once more. Histology showed no malignancy

Regarding the solely AI-driven models, the multiparametric AI was superior to biparametric AI with AUC of 0.68 vs. 0.64 (Fig. 3). Case examples of radiological/AI analysis of PI-RADS 3 + 1 lesions are depicted in Figs. 4 and 5.

A technical roadmap summarizing main aspects of methodology and results of our study can be found in Supplemental Fig. 2.

**Table 3:** Lasso penalized logistic regression analysis with remaining individual semantic and clinical features in different feature-based and feature-based/AI-enhanced models (lasso 1SE). In this approach, the number of features could be reduced significantly, with 3 radiologist features and 1 clinical feature remaining even in the same arrangement with regard to OR values in models 1–3. In model 4 the AI-feature mpCNN lesion-max was highest-ranked but the radiological semantic features “T2w configuration: irregular/microlobulated/spiculated”, “DWI suspicion: black and white” and “T2w texture: homogeneous, suspicious T2w hypointense” also remained with an OR > 1 in model 4. The semantic feature “leading sequence: T2w” and the clinical feature “log-transformed prostate volume” in model 4 were selected negatively correlated with sPC (OR < 1)

Model	Remaining features	OR
**Model 1**	**T2w configuration: irregular/microlobulated/spiculated**	**1.81**
(radiologist defined features only + clinical features)	T2w texture: homogeneous, suspicious T2w hypointense	1.11
	DWI suspicion: black and white	1.1
	Log-transformed prostate volume	0.89
**Model 2**	**T2w configuration: irregular/microlobulated/spiculated**	**2.1**
(radiologist defined features only excluding DCE features + clinical features)	T2w texture: homogeneous, suspicious T2w hypointense	1.26
	DWI suspicion: black and white	1.25
	Log-transformed prostate volume	0.76
**Model 3**	**T2w configuration: irregular/microlobulated/spiculated**	**1.81**
(Model 2 + bp CNN lesion-max)	T2w texture: homogeneous, suspicious T2w hypointense	1.11
	DWI suspicion: black and white	1.1
	Log-transformed prostate volume	0.89
**Model 4**	**mpCNN lesion-max**	**3.32**
(Model 1 + mp CNN lesion-max)	T2w configuration: irregular/microlobulated/spiculated	2.33
	DWI suspicion: black and white	1.18
	T2w texture: homogeneous, suspicious T2w hypointense	1.09
	Leading sequence: T2w	0.92
	Log-transformed prostate volume	0.81

Abbreviations: bp = biparametric, mp = multiparametric, OR = odds ratio, DCE = dynamic contrast enhancement, CNN = convolutional neural networks, DCE = dynamic contrast-enhanced, DWI = diffusion-weighted imaging

### Nomogram model - multivariable logistic regression model using selected predictors

Expert read based histopathological correlation of significant lexicon features is mentioned in Table 4. Based on the three selected semantic and the one clinical feature “prostate volume”, a nomogram for lesion-based prediction of sPC probability was build using a multivariable logistic regression model (as given in Supplemental Fig. 3). The predicted sPC probability can be derived from the following formula:

$$\:\text{Pr}\left(sPC\right)=\frac{1}{1+{e}^{-linear\:predictor}}$$

with.

linear predictor = + 3.454

+ 1.953 x (presence of feature “T2cims”)

+ 1.238 x (presence of feature “DWIbaw”)

+ 0.915 x (presence of feature “T2thsT2h”)

− 1.409 x (log-transformed prostate volume)

**Table 4:** Histopathological correlation of significant lexicon features (key features) based on expert consensus and systematic and targeted extended transperineal MRI/TRUS-fusion biopsy approach. Significant individual features are analyzed according to their ISUP score

Lesion based ISUP score	Feature 1 T2w configuration: irregular /microlobulated/ spiculated		Feature 2 T2w texture: homogeneous suspicious T2w hypointense		Feature 3 DWI suspicion: black and white	
Feature called (+) / not called (-) in expert consensus read (number of times)	+ (14)	− (80)	+ (27)	− (67)	+ (19)	− (75)
no cancer	2 (14.3%)	46 (57.5%)	8 (29.6%)	40 (59.7%)	5 (26.3%)	43 (57.3%)
ISUP 1	2 (14.3%)	17 (21.2%)	5 (18.5%)	14 (20.9)	3 (15.8%)	16 (21.3%)
ISUP 2	8 (57.1%)	11 (13.8%)	8 (29.6%)	11 (16.4%)	6 (31.6%)	13 (17.3%)
ISUP 3	1 (7.1%)	3 (3.8%)	3 (11.1%)	1 (1.5%)	3 (15.8%)	1 (1.3%)
ISUP 4	1 (7.1%)	1 (1.2%)	2 (7.4%)	0 (0%)	1 (5.3%)	1 (1.3%)
ISUP 5	0 (0%)	2 (2.5%)	1 (3.7%)	1 (1.5%)	1 (5.3%)	1 (1.3%)

## Discussion

**Fig. 6:**
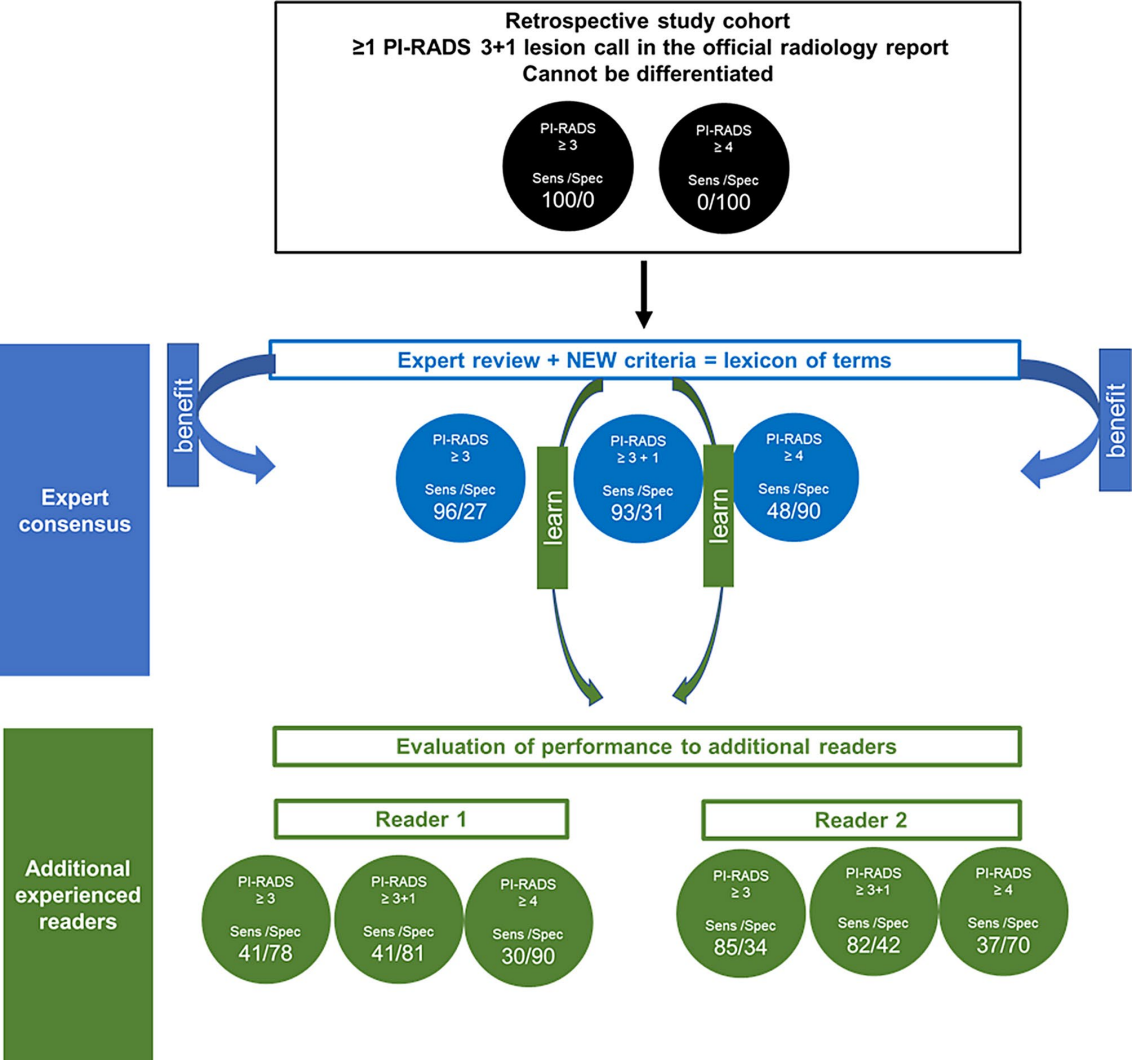
Risk stratification of clinically called PI-RADS 3 + 1 lesions based on expert review with generation of a feature catalog that was able to achieve risk stratification of lesions on basis of the consensus read. In a further step, learning and applying the expert criteria also enabled moderate to good risk stratification of other experienced prostate readers. Overall, the criteria make it possible to further differentiate an initially homogeneous, not further diversified lesion cohort

In this study, lexicon terms were defined and evaluated in consensus and subsequently utilized independently by two radiologists with different reading styles, demonstrating the ability to stratify lesions that clinically all received a DCE-upgraded PI-RADS 4 score (Fig. 6).

Based on expert consensus, five significant feature categories out of 14 were extracted including lesion size and two T2w and DWI categories each. Interestingly, DCE feature categories did not reach significance. This aligns with a previous study by Brancato et al. who highlighted the notable contributions of T2w and ADC radiomic features for stratification of equivocal PI-RADS 3 and upgraded PI-RADS 4 PZ lesions [29]. In univariate analysis of the individual features of the lexicon of terms, similar to feature categories, T2w and DWI features dominated compared to the DCE features (5 T2w features, 2 DWI features, 1 DCE feature) before multiplicity adjustment. The fact that the feature “T2cims” remained most significant after multiplicity adjustment indicates that a microlobulated or spiculated lesion appearance should imply a high level of suspicion in clinical assessment of PI-RADS 3 + 1 lesions. This complies with study findings of Rudolph et al., who reported rather high positive predictive values (PPV) for the PZ lexicon descriptors “irregular border” (43.8%), “spiculated border” (56.7%), and “lobulated shape” (56.8%). The marginally significant feature ”DWIbaw”, formally appears to be a PI-RADS 4 category descriptor, but lesions seem to have appeared less conspicuous during clinical interpretation, possibly due to their small size or superimposed inflammation. Lasso penalized regression models based on consensus read and additional clinical features as mentioned above did not show improved outcomes with the use of DCE lexicon terms when evaluated on a feature basis. Hence, the consensus radiologists did not gain a stratification benefit from consideration of DCE variables in the reading section. Prospective studies have already demonstrated the non-inferiority of biparametric MRI compared to mpMRI in detecting sPC while concurrently reducing the detection rates of non-sPC and the number of unnecessary biopsies [30, 31]. Regarding previous studies in larger screening cohorts without focus on PIRADS 3 + 1 lesions, a notable redundancy of DCE findings (i.e. focal lesion enhancement) compared to findings in T2w and DWI/ADC has been demonstrated. Visschere et al. showed a redundancy of DCE findings in 80.8% of patients, and recommend a multiparametric protocol only in patients with a PI-RADS score of 3 and without suspicious TZ lesions [32]. The latter statement contradicts the results of Tavakoli et al. who revealed that neither the quantitative nor the binary qualitative DCE analysis was beneficial in the prediction of clinical sPC in ADC PI-RADS 3 lesions and did not improve the distinction of lesions with intermediate suspicion [12]. Chatterjee et al. revealed that wedge-shaped PZ lesions, a common PI-RADS 3 + 1 morphologic appearance, do not benefit from utilization of early enhancement in distinguishing between benign and malignant lesions [14]. Aiming to improve MRI accuracy and reduce false positives, Messina proposed to biopsy PIRADS 3 and PIRADS 3up lesions only in cases of a PSA densitiy ≥ 0.15ng/ml^2^ [13]. However, the study protocol revealed several relevant limitations, i.e. a debatable PSA density cutoff and lack of biopsies in the PIRADS 3/3 + 1 subcohort without elevated PSA density [33] leading to a possibility of undetected prostate carcinomas in the cohort [34]. Thus, the role of PSA density in 3 + 1 lesions is still unclear. When we corrected for PSA density in our models (data not shown), the features “T2cims” and “T2thsT2h” were still selected into the models, showing that these imaging-based features are not just a surrogate of PSAD. In contrast, based on our own previous data, PI-RADS 3 lesions could be risk-stratified by adding a PSA density cutoff of < 0.1 ng/ml² with a 98% probability of excluding significant prostate cancer [35]. Of all considered clinical features in our study solely “log-transformed prostate volume” was selected into the models, however with lower contribution than the best semantic feature. This finding is plausible, as PSA elevation in patients with increased prostate volume may lead to the indication for MR evalution while BPH is an independent and non-malignant factor for PSA elevation.

In AI-based analysis, DCE resulted in a trend for improvement in diagnostic accuracy (ROC AUC increase from 0.64 to 0.68, non-significant in bootstrap AUC analysis), in agreement with previous studies [36, 37]. Matsuoka revealed improvements in specificity and positive predictive value within the PZ and fewer false-positives compared to biparametric AI approach [37] in multiparametric models. In our study, when radiologist-identified features were combined with AI predictions, the mpMRI AI model (using DCE information) provided a trend for a benefit to radiologists using lexicon terms, while the bpMRI model did not. Thus, while for radiologist assessment, DCE did not improve diagnostic performance, mpCNN may be able to provide complementary information.

The provided nomogram may provide decision support whether to perform prostate biopsy in the future, however requires confirmation in further studies.

Limitations of our study include the retrospective, single-institution, single-vendor setup, albeit in a high-volume imaging center with a high level of diagnostic experience and continued review of images in combination with extended histopathology results as discussed above. We are aware that retrospective studies carry the risk for underlying bias, which would not arise in prospective analysis. We have tried to design the study setup and the inclusion criteria in such a manner that the risk for bias was removed as best we could. There was no specific patient selection for this study than including prostate MRI examinations within a continuous time frame of 29 months that had at least one peripheral PI-RADS 3 + 1 lesion in the official clinical radiology report. We also assume that the study design was chosen carefully enough to avoid the risk for recall bias i.e. by long-enough wash-out periods comparable to those found in the literature. Multi-centric and multi-vendor study settings are certainly advantageous and necessary to transfer study results into clinical practice. Those setups are widely discussed in the AI community and primarily serve to further investigate performances of models that have already shown good results in mono-institutional studies. Given the scarce evidence of studies addressing the topic of PI-RADS 3 + 1, we believe our study highlights open questions which have not been addressed to more extent so far, even in a mono-institutional study setup. We even believe that the homogeneity of data can actually be of advantage. Multi-vendor and multi-institution studies would in turn mean added variability and data heterogeneity that would increase the number of prostate MRI cases to be assessed to discover potential underlying relationships. Furthermore an even larger sample size would have been beneficial e.g. with regard to even more robust models and statistical power. However, it should be noted that the scarcity of PI-RADS 3 + 1 cases in itself limits the available data. Otherwise, the sample size in our study enabled a detailed evaluation of PI-RADS 3 + 1 lesions by a total of 4 experienced radiologists. We utilized in-house developed and trained deep learning pipelines, which have not been validated in many institutions. However, validation has been performed to extra-institutional data, both using public datasets [23] and bi-institutionally [19].

In conclusion, we addressed a large number of clinical PI-RADS 3 + 1 lesions by re-evaluating them both by expert consensus and independent board-certified radiologist re-assessment, thereby establishing radiological and clinical key features for nomogram building that exhibit potential to aid in improved sPC detection. By comparing them to deep-learning performance, we find no significant contribution of DCE, although trends are discovered suggesting that deep learning may contribute only to radiological feature assessment when multiparametric information, but not when only biparametric information is utilized in DL models, findings that require further evaluation in larger cohorts. We believe that this study performed in a homogeneous setting provides important novel insights into the assessment of PI-RADS 3 + 1 lesions that may inform larger future multi-centric, multi-vendor studies.

## Supplementary Information

Below is the link to the electronic supplementary material.

Supplementary Material 1

## Data Availability

The data used in this manuscript are subject to local privacy and data protection laws and not publicly available. Requests for anonymized data review can be directed to the authors and will be reviewed in co-operation with the responsible data protection officers.

## Abbreviations

adj.: Adjusted
ADC: Apparent diffusion coefficient
AI: Artificial intelligence
AUC: Area under the receiver operating characteristics curve
bpMRI: Biparametric magnetic resonance imaging
CNN: Convolutional neural network
(c)sPC: (clinically) significant prostate cancer
DCE: Dynamic contrast enhancement
DCEltetr: DCE lesion timing earlier than rest
DL: Deep learning
DWI: Diffusion-weighted images
DWIbaw: DWI suspicion: black and white
DWIsADCsBVALs: DWI suspicion: ADC subtle/BVAL subtle
ESUR: European Society of Urogenital Radiology
ICC: Intraclass correlation coefficient
ISUP: International Society of Urological Pathology
lsT2: Leading sequence: T2w
MR: Magnetic resonance
MRI: Magnetic resonance imaging
mpMRI: Multiparametric magnetic resonance imaging
nnUNet: no new U-Net
OR: Odds ratio
PC: Prostate cancer
PI-RADS: Prostate Imaging Reporting and Data System
PI-RADS: 3up upgraded PI-RADS 3 lesion, alternative term to PI-RADS 3 + 1
PPV: Positive predictive value
PSA(D): Prostate-specific antigen (density)
PZ: Peripheral zone
R1: Reader 1
R2: Reader 2
ROC: Receiver operating characteristics
SE: Standard error
sPC: Significant prostate cancer
T2w: T2-weighted
T2cims: T2w configuration: irregular/microlobulated/spiculated
T2ccsbnpw: T2w configuration: concave sharp borders no perfect wedge
T2thsT2h: T2w texture: homogeneous suspicious T2w hypointense
T2tlorT2b: T2w texture: linear or reticular T2w bright
TRUS: Transrectal ultrasound
TZ: Transitional zone
