# Supplementary material for: Improving risk stratification of PI-RADS 3 + 1 lesions of the peripheral zone: expert lexicon of terms, multi-reader performance and contribution of artificial intelligence
Source: Cancer Imaging. 2025 Aug 19;25:102. doi: 10.1186/s40644-025-00916-7 (PMC12366217; doi:10.1186/s40644-025-00916-7)

**Supplemental Section**

**Supplemental Material 1:**

T2w imaging included a sagittal T2w BLADE (echo time (TE) 105mm, repetition time (TR) 5000ms, field of view (FOV) 200mm and 3mm slice thickness), a tranversal T2w TSE (TE 145mm, TR 8080ms, FOV 200mm, slice thickness 3mm) and a coronal T2w TSE (TE 145mm, TR 8000ms, FOV 200mm, slice thickness 3mm). DWI imaging contained an EPI SPAIR sequence (spectral adiabatic inversion recovery) with b-values of 50,500, 1000 and 1500 (TE 48ms, TR 3300ms, FOV 280ms, slice thickness 3mm). Dynamic contrast-enhanced imaging (DCE) contained a transversal dynamic T1w TWIST (TE 2,1ms, TR 4,45, FOV 300mm, 35 measurements, 5 seconds temporal resolution). T1w imaging included a native T1w TSE DIXON (TE 13ms, TR 773ms, FOV 360mm, slice thickness 4mm), a contrast-enhanced transversal T1w VIBE DIXON (TE 2,4ms / 3,69 ms, TR 5,4ms, FOV 380mm, slice thickness 1,2mm) and a contrast-enhanced coronal T1w TSE DIXON (TE 13ms, TR 773, FOV 360mm, slice thickness 4mm).

**Supplemental Material 2:**

Two patients who had received a total of 35 or 37 biopsy cores respectively were excluded from the statistical analysis as the exact numbers of targeted lesion cores in different lesions (case 1) and the exact number of systematic and targeted cores (case 2) could not be determined in pathological or surgical report retrospectively.

**Supplemental Table 1:** Expert consensus read features and their relation to PI-RADS criteria.

| **Feature name/description** | **Features in comparison to PI-RADS v2.1. (N=new, O= overlap, E=existing)** | **Further explanation due to PI-RADS V2.1.** |
| --- | --- | --- |
| Qualitative size: invisible/very small | N | - |
| Qualitative size: regular | N | - |
| Qualitative size: very large | N | - |
| Leading sequence: ADC/DWI all | E | for PZ lesions |
| Leading sequence: BVAL 1500 | N | - |
| Leading sequence:  DCE | N | - |
| Leading sequence:  T2w | O | Only for TZ lesions, not for PZ lesions |
| Localization: within PZ | N | PZ lesions not further subdivided regarding localization in PZ in PI-RADS |
| Localization: subcapsular | N |  |
| Localization: interface | N |  |
| T2w configuration: classic wedge shape, sharp borders | E | wedge as PI-RADS descriptor |
| T2w configuration: concave sharp borders, no perfect wedge | N | - |
| T2w configuration: focality within diffuse changes | O | partly  (focal abnormality as a PI-RADS descriptor) |
| T2w configuration: inflammation configuration reticular | N | - |
| T2w configuration: irregular/ microlobulated/spiculated | O (*micro*lobulated new (more detailed description)) | irregular, spiculated and lobulated are PI-RADS descriptors |
| T2w configuration: clearly benign findings | N | - |
| T2w configuration: macrolobulated/polygonal | O (*macro*lobulated new (more detailed description), polygonal new feature) | lobulated as PI-RADS descriptor |
| T2w configuration: patchy/nonfocal | N | - |
| T2w configuration: punctate | N | - |
| T2w configuration: round/oval | E | round and oval are PI-RADS descriptors |
| T2w texture: encapsulated nodule without T2w bright spots | O | Partly (encapsulated as PI-RADS descriptor) |
| T2w texture: hidden/obscured in/by inflammation | O | Partly (obscured as PI-RADS descriptor) |
| T2w texture: homogeneous suspicious T2w hypointense | O | Partly (T2w hypointense as PI-RADS descriptor) |
| T2w texture: linear or reticular T2w bright | O | Partly (linear as PI-RADS descriptor) |
| T2w texture: mild to moderate or heterogeneous T2w hypointense | O | Partly (T2w hypointense as PI-RADS descriptor, in the description for PI-RADS 3 PZ lesion (T2w): “heterogeneous signal intensity or non-circumscribed, rounded moderate hypointensity) |
| T2w texture: T2w bright spots | N | - |
| T2w texture: very small hypointense | N | - |
| DWI configuration: confluent microfoci | N | - |
| DWI configuration: hidden/invisible | O | Partly (obscured as PI-RADS descriptor) |
| DWI configuration: lobulated | E | lobulated as PI-RADS descriptor |
| DWI configuration: patchy | N | - |
| DWI configuration: polygonal | N | - |
| DWI configuration: punctate | N | - |
| DWI configuration: round/oval | E | round and oval are PI-RADS descriptors |
| DWI suspicion: ADC or BVAL clear, corresponding BVAL or ADC no correlate or subtle | O | - |
| DWI suspicion: ADC subtle, BVAL subtle | E | exact wording for PI-RADS 3 lesions in the PZ due to DWI characteristic in the PI-RADS manual 2.1.: “focal (discrete and different from the background) hypointense on ADC and/or focal hyperintense on high b-value DWI; may be markedly hypointense on ADC or markedly hyperintense on high b-value DWI, but not both” |
| DWI suspicion: black and white | E |  |
| DCE configuration: hidden | O | Partly (obscured as a PI-RADS descriptor) |
| DCE configuration: patchy | N | - |
| DCE configuration: polygonal | N | - |
| DCE configuration: punctate | N | - |
| DCE configuration: round/oval | E | round and oval are PI-RADS descriptors |
| DCE margin: hidden | O | Partly (obscured as PI-RADS descriptor) |
| DCE margin: sharp | O | Partly (circumscribed as PI-RADS descriptor) |
| DCE margin: unsharp | O | Partly (non-circumscribed as PI-RADS descriptor) |
| DCE lesion timing: earlier than rest | O | Partly (exact wording for DCE positive lesions due to PI-RADS 2.1. manual: “focal, and; earlier than or contemporaneously with enhancement of adjacent normal  prostatic tissues, and; corresponds to suspicious finding on T2W and/or DWI” |
| DCE lesion timing: equal to TZ or inflammation | O | Partly (exact wording for DCE positive lesions due to PI-RADS 2.1. manual: “focal, and; earlier than or contemporaneously with enhancement of adjacent normal  prostatic tissues, and; corresponds to suspicious finding on T2W and/or DWI”) |
| DCE lesion timing: later than central enhancement | O | Partly (exact wording for DCE negative lesion due to PI-RADS 2.1. manual: no early or contemporaneous enhancement;  or diffuse multifocal enhancement NOT corresponding to a focal finding on T2W  and/or DWI or focal enhancement corresponding to a lesion demonstrating features  of BPH on T2WI (including features of extruded BPH in the PZ”) |
| T2w to DWI match: match | O | - |
| T2w to DWI match: mismatch | O | - |
| T2w to DCE match: match | O | - |
| T2w to DCE match: mismatch | O | - |
| T2w biplanar detection: yes | O | - |
| T2w biplanar detection:  No | O | - |
| DCE match: DCE clearly visible | E | exact wording for DCE positive lesions due to PI-RADS 2.1. manual: “focal, and; earlier than or contemporaneously with enhancement of adjacent normal  prostatic tissues, and; corresponds to suspicious finding on T2W and/or DWI”) |
| DCE match: DCE subtle | E | - |
| DCE match: DCE hidden/partly hidden in inflammation | N | - |
| DCE match: DCE negative | E | exact wording for DCE negative lesion due to PI-RADS 2.1. manual: no early or contemporaneous enhancement;  or diffuse multifocal enhancement NOT corresponding to a focal finding on T2W  and/or DWI or focal enhancement corresponding to a lesion demonstrating features  of BPH on T2WI (including features of extruded BPH in the PZ” |
| DCE match: DCE very brisk | O | Partly (exact wording for DCE positive lesions due to PI-RADS 2.1. manual: “focal, and; earlier than or contemporaneously with enhancement of adjacent normal  prostatic tissues, and; corresponds to suspicious finding on T2W and/or DWI”)) |

**Supplemental Table 2:** Lesional PI-RADS decisions in expert consensus read and single reads (Expert consensus vs. single reader 1) for determination of inter-rater agreement.

| PI-RADS scoring  single Reader 1  Expert Consensus  Results (lesional PI-RADS score) | 2 | 3 | 4 | 5 | Sum |
| --- | --- | --- | --- | --- | --- |
| 2 | 18 | 0 | 1 | 0 | 19 |
| 3 | 3 | 1 | 0 | 0 | 4 |
| 4 | 46 | 1 | 17 | 0 | 64 |
| 5 | 1 | 0 | 2 | 4 | 7 |
| Sum | 68 | 2 | 20 | 4 | 94 |

**Supplemental Table 3:** Lesional PI-RADS decisions in expert consensus read and single reads (consensus vs. single reader 2) for determination of inter-rater agreement.

| PI-RADS scoring  single Reader 2  Expert Consensus  Results (lesional PI-RADS score) | 2 | 3 | 4 | 5 | Sum |
| --- | --- | --- | --- | --- | --- |
| 2 | 9 | 0 | 10 | 0 | 19 |
| 3 | 3 | 1 | 0 | 0 | 4 |
| 4 | 14 | 4 | 45 | 1 | 64 |
| 5 | 1 | 1 | 2 | 3 | 7 |
| Sum | 27 | 6 | 57 | 4 | 94 |

**Supplemental Table 4:** Lesional PI-RADS decisions between single reads (single reader 1 vs. single reader 2) for determination of inter-rater agreement.

| PI-RADS scoring  single Reader 2  PI-RADS scoring  singe Reader 1 | 2 | 3 | 4 | 5 | Sum |
| --- | --- | --- | --- | --- | --- |
| 2 | 25 | 3 | 40 | 0 | 68 |
| 3 | 1 | 1 | 0 | 0 | 2 |
| 4 | 1 | 1 | 16 | 2 | 20 |
| 5 | 0 | 1 | 1 | 2 | 4 |
| Sum | 27 | 6 | 57 | 4 | 94 |

**Supplemental Table 5:** Univariable tests for association with sPC of pre-selected features of the PI-RADS 3+1 lesion feature set based on consensus read.

| **Variable** | **OR** | **p-value** | **adj. p-value** | **Sens (95%CI)** | **Spec (95%CI)** |
| --- | --- | --- | --- | --- | --- |
| T2w configuration: irregular/microlobulated/spiculated | 9.0 (2.3-44.3) | <0.001 | 0.016 | 37 (19-58) | 94 (85-98) |
| T2w texture: homogeneous, suspicious T2w hypointense | 4.4 (1.5-13.1) | 0.003 | 0.046 | 52 (32-71) | 81 (69-89) |
| DWI suspicion: black and white | 5.0 (1.5-16.9) | 0.004 | 0.046 | 41 (22-61) | 88 (78-95) |
| DWI suspicion: ADC subtle/BVAL subtle | 0.3 (0.1-0.8) | 0.009 | 0.086 | 59 (39-78) | 72 (59-82) |
| Leading sequence: T2w | 0.3 (0.1-0.8) | 0.012 | 0.096 | 74 (54-89) | 55 (43-67) |
| DCE lesion timing: earlier than rest | 4.8 (1.2-21.0) | 0.018 | 0.113 | 30 (14-50) | 92 (82-97) |
| T2w texture: linear or reticular T2w bright | 0.1 (0.0-0.9) | 0.020 | 0.113 | 4 (0-19) | 76 (64-86) |
| T2w configuration: concave sharp borders no perfect wedge | 0.0 (0.0-0.9) | 0.030 | 0.147 | 0 (0-13) | 84 (73-92) |
| DCE configuration: round/oval | 2.3 (0.8-6.4) | 0.094 | 0.320 | 48 (29-68) | 71 (59-82) |
| DWI configuration: polygonal | 0.2 (0.0-1.3) | 0.100 | 0.320 | 4 (0-19) | 82 (71-90) |
| DCE match: DCE subtle | 0.3 (0.1-1.2) | 0.105 | 0.320 | 11 (2-29) | 71 (59-82) |
| DWI configuration: round/oval | 2.2 (0.8-6.2) | 0.106 | 0.320 | 56 (35-75) | 64 (52-76) |
| DCE lesion timing: equal to TZ or inflammation | 0.4 (0.1-1.4) | 0.107 | 0.320 | 67 (46-83) | 17 (9-29) |
| DCE configuration: patchy | 0.4 (0.1-1.3) | 0.124 | 0.345 | 15 (4-34) | 68 (56-79) |
| Leading sequence: DCE | 2.8 (0.6-13.4) | 0.144 | 0.366 | 19 (6-38) | 93 (83-98) |
| DCE margin: sharp | 2.0 (0.7-5.5) | 0.163 | 0.366 | 52 (32-71) | 65 (52-76) |
| DCE configuration: hidden | 0.2 (0.0-1.7) | 0.167 | 0.366 | 4 (0-19) | 85 (74-92) |
| Qualitative size: invisible/ very small | 0.2 (0.0-1.5) | 0.169 | 0.366 | 4 (0-19) | 84 (73-92) |
| T2w to DWI match: match | 0.5 (0.1-1.9) | 0.216 | 0.431 | 78 (58-91) | 12 (5-22) |
| Leading sequence: ADC/DWI all | 1.9 (0.6-5.3) | 0.221 | 0.431 | 41 (22-61) | 73 (61-83) |
| DWI configuration: patchy | 0.4 (0.1-1.6) | 0.256 | 0.458 | 11 (2-29) | 76 (64-86) |
| T2w texture: T2w bright spots | 0.4 (0.1-1.8) | 0.258 | 0.458 | 11 (2-29) | 78 (66-87) |
| T2w configuration: patchy/nonfocal | 0.3 (0.0-2.0) | 0.272 | 0.461 | 4 (0-19) | 87 (76-94) |
| DCE match: DCE clearly visible | 1.6 (0.6-4.5) | 0.362 | 0.568 | 59 (39-78) | 53 (40-65) |
| DCE margin: unsharp | 0.6 (0.2-1.7) | 0.364 | 0.568 | 37 (19-58) | 52 (39-64) |
| T2w biplanar detection: yes | 1.9 (0.5-8.8) | 0.412 | 0.618 | 85 (66-96) | 25 (16-37) |
| DCE configuration: polygonal | 1.6 (0.4-5.6) | 0.545 | 0.788 | 22 (9-42) | 85 (74-92) |
| Qualitative size: regular | 0.8 (0.2-2.6) | 0.594 | 0.823 | 74 (54-89) | 21 (12-33) |
| Localization: within PZ | 0.8 (0.3-2.2) | 0.633 | 0.823 | 63 (42-81) | 31 (21-44) |
| Leading sequence: BVAL 1500 | 1.5 (0.3-6.5) | 0.724 | 0.823 | 15 (4-34) | 90 (80-96) |
| Localization: interface | 1.3 (0.3-5.4) | 0.738 | 0.823 | 15 (4-34) | 88 (78-95) |
| DCE margin: hidden | 0.6 (0.1-2.7) | 0.750 | 0.823 | 11 (2-29) | 83 (72-91) |
| DWI suspicion: ADC or BVAL clear corresponding BVAL or ADC no correlate or subtle | 1.2 (0.3-4.1) | 0.771 | 0.823 | 19 (6-38) | 84 (73-92) |
| T2w configuration: round/oval | 1.2 (0.3-4.1) | 0.771 | 0.823 | 19 (6-38) | 84 (73-92) |
| DCE match: DCE hidden/ partly hidden in inflammatory | 0.8 (0.2-3.0) | 0.772 | 0.823 | 15 (4-34) | 82 (70-90) |
| T2w configuration: macrolobulated/polygonal | 1.3 (0.4-4.4) | 0.773 | 0.823 | 22 (9-42) | 82 (71-90) |
| Localization: subcapsular | 1.2 (0.3-3.9) | 0.781 | 0.823 | 22 (9-42) | 81 (69-89) |
| T2w to DCE match: match | 1.1 (0.3-4.4) | 1.000 | 1.000 | 81 (62-94) | 20 (11-32) |
| T2w texture: mild to moderate or heterogeneous T2w hypointense | 0.8 (0.2-3.0) | 1.000 | 1.000 | 15 (4-34) | 82 (71-90) |

Abbreviations: OD= odds ratio, adj.=adjusted, sens=sensitivity, spez= specificity, T2w= T2w weighted, DWI= diffusion weighted imaging, ADC= apparent diffusion coefficient, BVAL=b-value, DCE=dynamic contrast-enhanced, PZ=peripheral zone

**Supplemental Figure 1:** Patient selection flow chart, *previously published study cohort as in the following reference: *Schrader A, Netzer N, Hielscher T, et al. Prostate cancer risk assessment and avoidance of prostate biopsies using fully automatic deep learning in prostate MRI: comparison to PI-RADS and integration with clinical data in nomograms. Eur Radiol. 2024 Jul 2. doi: 10.1007/s00330-024-10818-0.*


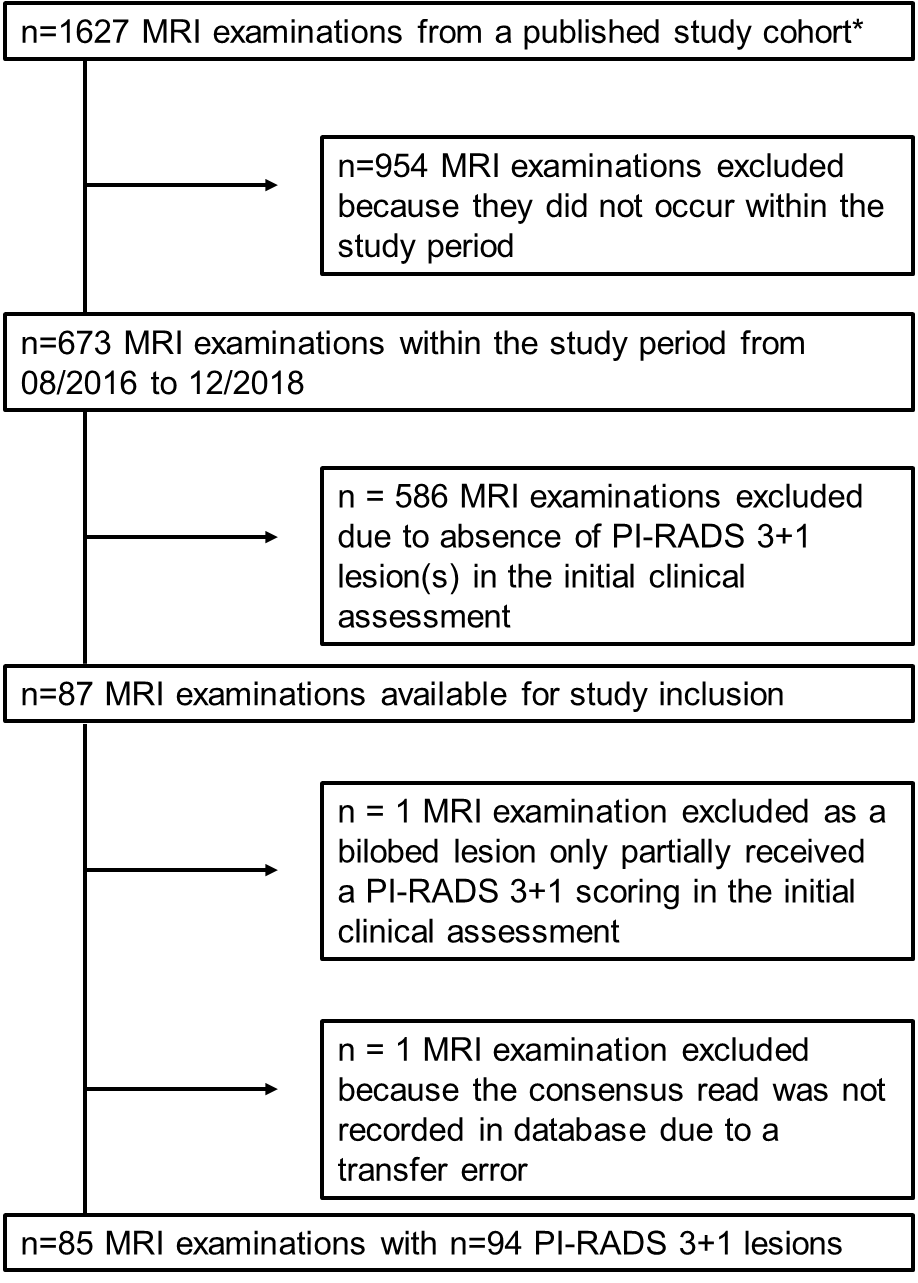


n = 1 MRI examination excluded because the consensus read was not recorded in database due to a transfer error

n=85 MRI examinations with n=94 PI-RADS 3+1 lesions

**Supplemental Figure 2:**

Technical roadmap of our study approach for risk-stratification of peripheral PI-RADS 3+1 lesions with chronological steps of study methodology. Two prostate experts created features for semantic description of PI-RADS 3+1 lesions (step 1 and 2) and subsequent univariate analysis was performed for sPC prediction (step 3). By this, 3 features (as named under step 4) were significant. For feature reduction additional lasso penalized logistic regression models including clinical features (PSA, patient’s age, histological results of any previous biopsy, log-transformed prostate volume) with and without AI were conducted as well (step 5). The selected imaging features in models 1 to 4 remained identical with prostate volume always being selected as additional predictor. A nomogram was built subsequently based upon the most relevant semantic features and the clinical feature “prostate volume” as mentioned in step 6.


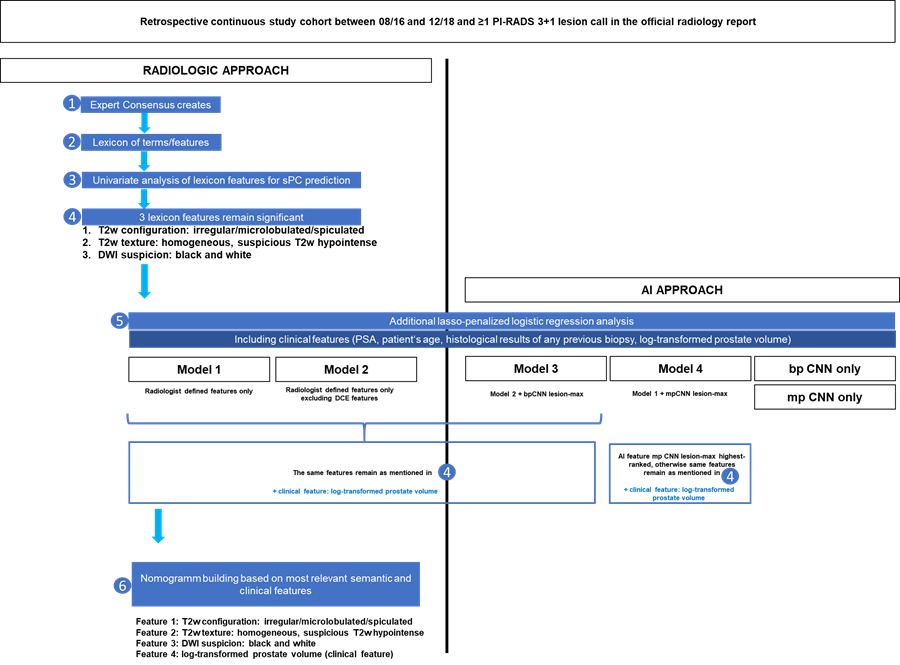


**Supplemental Figure 3:** Nomogram for predicted sPC probability based on significant features in clinical-feature-enriched lasso penalized logistic regression models in our study. By adding up the individual point values of existing features in a peripheral PI-RADS 3+1 lesion (0= not exisiting, 1=existing) the total points can be determined. The predicted probability for sPC can then be read from the nomogram.


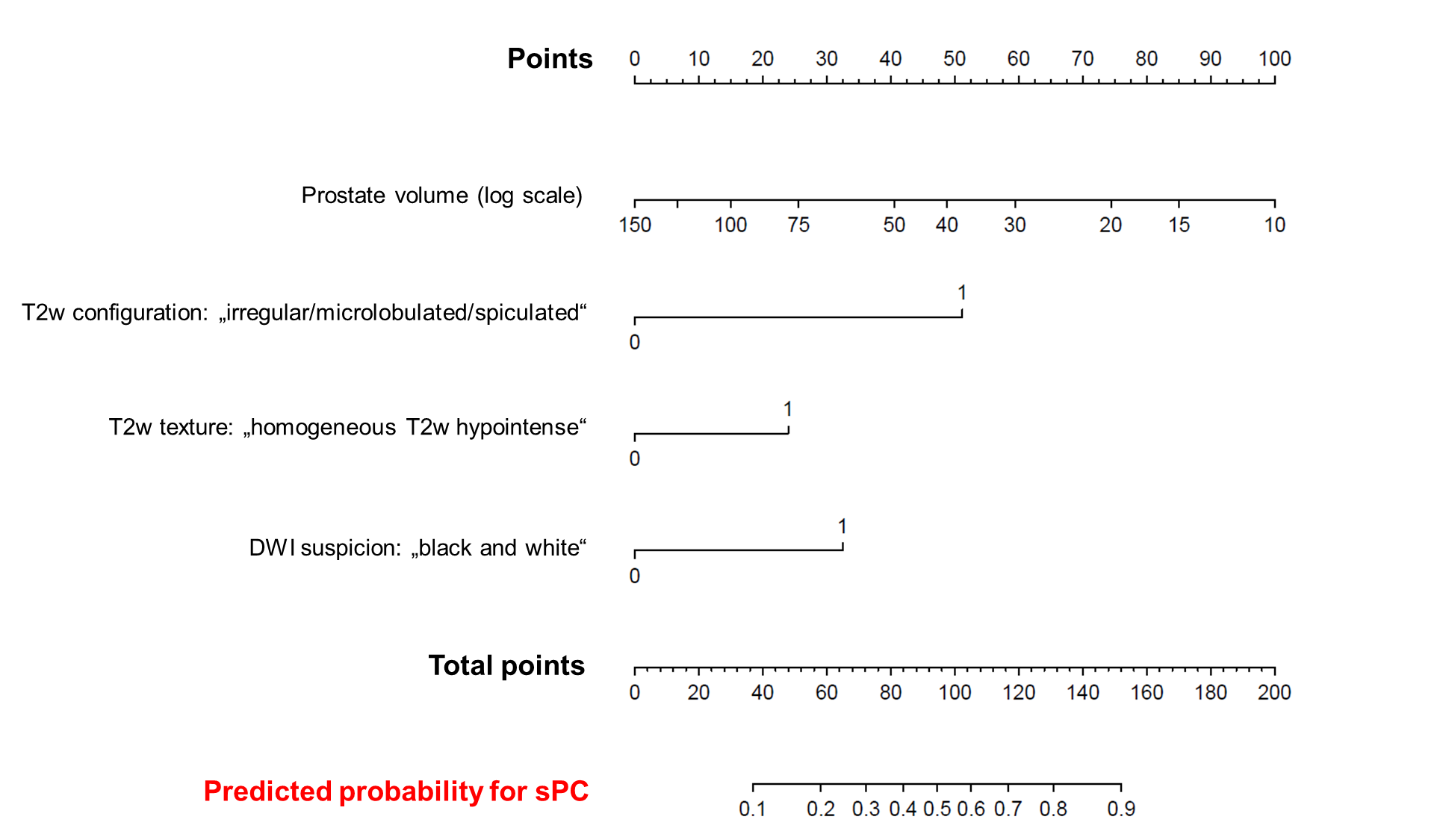

Supplement: Supplementary file 1 — Supplementary Material 1 [file 40644_2025_916_MOESM1_ESM.docx]
